# A novel rabbit model of Duchenne muscular dystrophy generated by CRISPR/Cas9

**DOI:** 10.1242/dmm.032201

**Published:** 2018-06-04

**Authors:** Tingting Sui, Yeh Siang Lau, Di Liu, Tingjun Liu, Li Xu, Yandi Gao, Liangxue Lai, Zhanjun Li, Renzhi Han

**Affiliations:** 1 Jilin Provincial Key Laboratory of Animal Embryo Engineering, Jilin University, Changchun, 130062, China; 2 Department of Surgery, Davis Heart and Lung Research Institute, Biomedical Sciences Graduate Program, Biophysics Graduate Program, The Ohio State University Wexner Medical Center, Columbus, OH 43210, US

**Keywords:** CRISPR, Cardiomyopathy, Dystrophin, Gene editing, Muscular dystrophy, Rabbit

## Abstract

Duchenne muscular dystrophy (DMD) is an X-linked muscle-wasting disorder caused by mutations in the dystrophin gene, with an incidence of 1 in 3500 in new male births. *Mdx* mice are widely used as an animal model for DMD. However, these mice do not faithfully recapitulate DMD patients in many aspects, rendering the preclinical findings in this model questionable. Although larger animal models of DMD, such as dogs and pigs, have been generated, usage of these animals is expensive and only limited to several facilities in the world. Here, we report the generation of a rabbit model of DMD by co-injection of Cas9 mRNA and sgRNA targeting exon 51 into rabbit zygotes. The *DMD* knockout (KO) rabbits exhibit the typical phenotypes of DMD, including severely impaired physical activity, elevated serum creatine kinase levels, and progressive muscle necrosis and fibrosis. Moreover, clear pathology was also observed in the diaphragm and heart at 5 months of age, similar to DMD patients. Echocardiography recording showed that the DMD KO rabbits had chamber dilation with decreased ejection fraction and fraction shortening. In conclusion, this novel rabbit DMD model generated with the CRISPR/Cas9 system mimics the histopathological and functional defects in DMD patients, and could be valuable for preclinical studies.

This article has an associated First Person interview with the first author of the paper.

## Abstract (precis)

**Summary:** The DMD KO rabbit engineered by CRISPR genome editing faithfully recapitulates the DMD pathologies, and could be a valuable tool for basic and translational studies to combat this disease.

## INTRODUCTION

Duchenne muscular dystrophy (DMD) is a fatal X-linked recessive disease characterized by progressive muscle weakening and wasting. DMD patients usually lose the ability to walk between 10 and 12 years of age and die of respiratory and/or cardiac failure by the age of 20-30 years (Spurney, 2011). DMD affects ∼1 in 3500 male births according to newborn screening (Mendell and Lloyd-Puryear, 2013; Mendell et al., 2012).

DMD is caused by mutations in the *DMD* gene, which encodes dystrophin protein (Bonilla et al., 1988; Hoffman et al., 1987). The *DMD* gene consists of 79 exons on the X chromosome (Hoffman et al., 1987), and mutations occur in any of these exons with the common hotspots in exons 3-7 and exons 45-55. The mutations include various forms: large deletion (68%), large duplication (11%), point mutations (11%) and small insertion/deletion (7%) (Bladen et al., 2015). These mutations cause frameshift and/or premature stop codon formation in the *DMD* gene, thus disrupting the expression of dystrophin and leading to the development of DMD (Beggs et al., 1991). Currently, there is no effective therapeutic treatment available for DMD.

At present, several animal models of DMD presenting the dystrophic phenotype are available, including mouse (Bulfield et al., 1984; Chapman et al., 1989; Sicinski et al., 1989), dog (Baltzer et al., 2007; Cooper et al., 1988; Jones et al., 2004; Sharp et al., 1992; Smith et al., 2011) and pig (Klymiuk et al., 2013). Many of the pathogenesis and preclinical studies were initially carried out in *Mdx* (*Dmd*) mice, a widely used animal model of DMD. However, the *Mdx* mice do not recapitulate human DMD patients in many aspects. For example, the phenotype of *Mdx* mice is much milder compared with that of DMD patients, with almost a normal life span (Chamberlain et al., 2007), although they exhibit muscular dystrophy (Bulfield et al., 1984; Chapman et al., 1989; Sicinski et al., 1989). This could be part of the reason for the poor translation of the findings achieved with these animals. Golden retriever muscular dystrophy (GRMD) dogs (Baltzer et al., 2007; Cooper et al., 1988; Jones et al., 2004; Sharp et al., 1992; Smith et al., 2011), on the other hand, show severe muscular dystrophy pathology, lethal respiratory distress and cardiomyopathy, resembling DMD patients. Dystrophin-deficient pigs were generated by deleting exon 52 in pig fibroblasts followed by nuclear transfer (Klymiuk et al., 2013). These pigs exhibit many features of muscular dystrophy including elevated serum creatine kinase, impaired mobility and progressive dystrophic changes in skeletal muscle (Klymiuk et al., 2013). Interestingly, pathological alterations were not observed in the heart of these pigs; however, some DMD pigs also die shortly after birth (Klymiuk et al., 2013; Yu et al., 2016). Although GRMD dogs and DMD pigs resemble human DMD patients better than *Mdx* mice do, the cost associated with the usage of these large animals poses a big hurdle for many laboratories.

Therefore, there is a need to develop DMD models in other species, which must recapitulate human DMD patients with reasonable costs for maintenance in ordinary laboratories. Of note, the rabbit shares more similarities with humans in terms of physiology, anatomy and genetics than does the mouse (Wang et al., 2014), and has been extensively used as an appropriate model for cardiovascular and metabolic disease research (Bősze et al., 2003). Lower maintenance cost and shorter gestational duration makes the rabbit a superior model compared with dogs or pigs.

In this study, we established a novel DMD rabbit model by cytoplasm microinjection of Cas9 mRNA and single guide RNA (sgRNA). These clustered regularly interspaced short palindromic repeats (CRISPR)/Cas9 knockout (KO) rabbits showed many features of muscular dystrophy including elevated serum creatine kinase (CK), muscle necrosis and regeneration, impaired mobility, and increased fibrosis in the heart and bladder. This novel DMD rabbit model could be a valuable resource for DMD research and preclinical studies.

## RESULTS

### Generation of *DMD* KO rabbits using CRISPR/Cas9

**Fig. 1.**
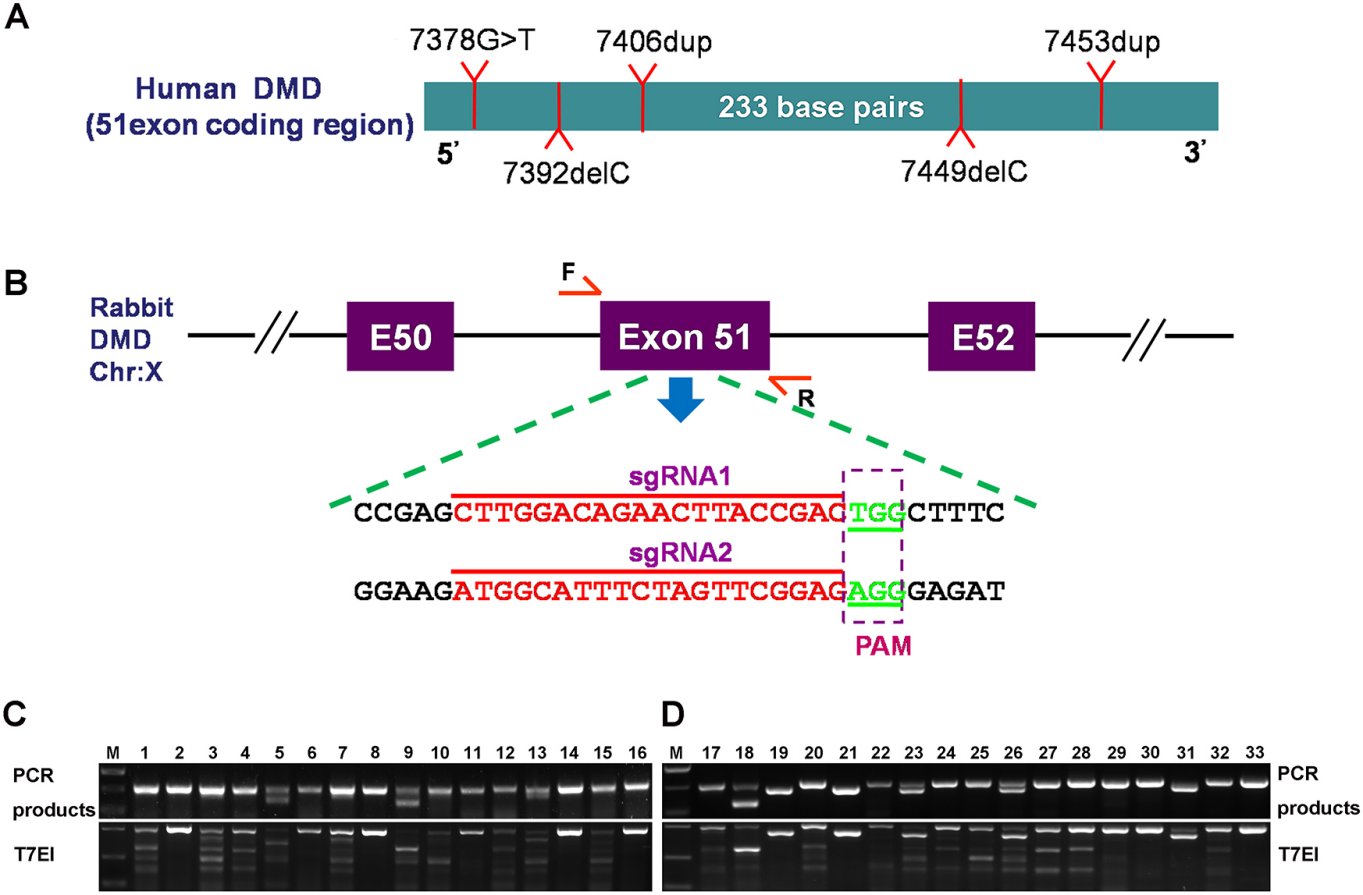
**Generation of *DMD* KO rabbits using CRISPR/Cas9.** (A) The common mutations in exon 51 in human DMD patients. (B) Schematic diagram of two sgRNA target sites located in exon 51 of the rabbit *DMD* locus. *DMD* exons are indicated by purple boxes; target sites of the two sgRNA sequences (sgRNA1 and sgRNA2) are highlighted in red; protospacer-adjacent motif (PAM) sequences are highlighted in green. (C,D) Mutation detection by the T7E1 cleavage assay in pups 1-16 (C) and 17-33 (D). M, the DNA ladder (DL2000).

In order to disrupt the open reading frame (ORF) of *DMD* in rabbits, we designed a pair of sgRNAs targeting exon 51, which is commonly mutated in human DMD patients (Fig. 1A,B). To test the efficiency of CRISPR/Cas9-mediated gene targeting of *DMD* in zygotes, the mixed Cas9 mRNA and sgRNAs were microinjected into the zygotes, and cultured until the blastocyst stage. As shown in Table 1, 79.6% of injected embryos (*n*=123) developed into the blastocyst stage, among which 78.0% carried mutations in *DMD* at the target sites. There were no significant differences in the developmental rate between the noninjected embryos and microinjected embryos (*P*>0.05). These results demonstrated that the dual sgRNA-directed CRISPR/Cas9 system is efficient for generation of mutations in the *DMD* gene in rabbit zygotes.

**Table 1.**
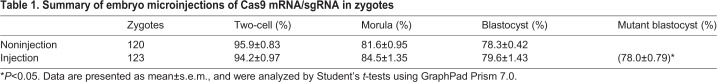
**Summary of embryo microinjections of Cas9 mRNA/sgRNA in zygotes**

In order to generate *DMD* KO rabbits, a total of 128 injected zygotes were transferred into the oviducts of four surrogate rabbits. All surrogates were pregnant to term and produced 33 live pups (Table 2). The genomic DNA from each pup was isolated, and mutations were determined by T7 endonuclease I (T7E1) assay and Sanger sequencing of the PCR products near the target sites. As shown in Fig. 1C and Fig. S1, 26 of the 33 (78.8%) newborn pups carried a *DMD* mutation, and 22 of them (84.6%) carried the biallelic *DMD* mutations. In order to examine the off-target effects in these *DMD* KO rabbits, the PCR products of the top ten potential off-target sites were subjected to Sanger sequencing and T7E1 cleavage assay. No off-target mutations were detected at these potential sites in the *DMD* KO rabbits (Fig. S2).

**Table 2.**
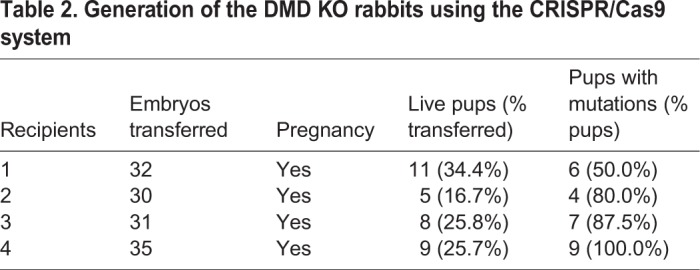
**Generation of the DMD KO rabbits using the CRISPR/Cas9 system**

### Disruption of dystrophin expression in the *DMD* KO rabbits

**Fig. 2.**
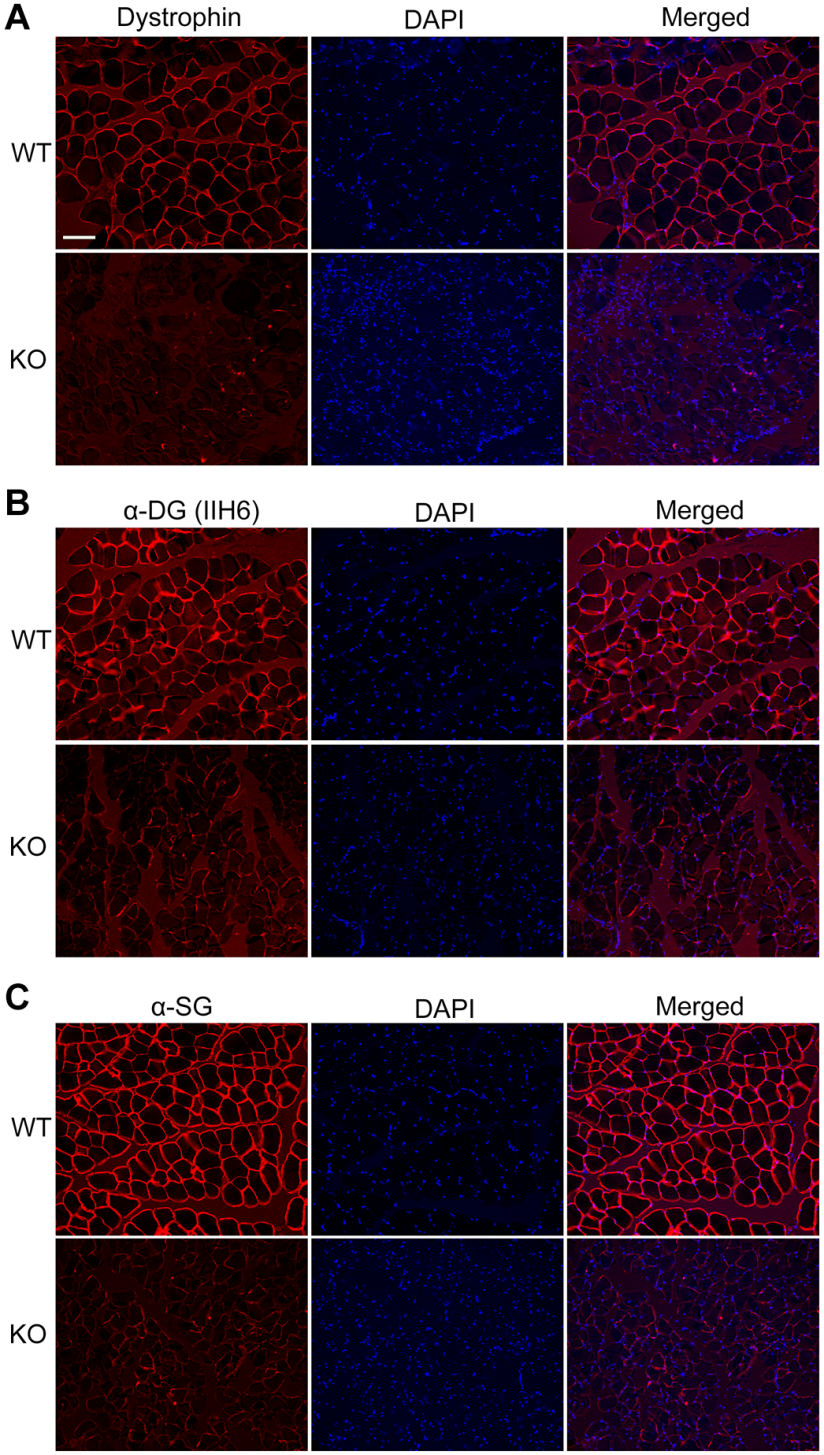
**Disruption of dystrophin expression in the skeletal muscle of *DMD* KO rabbits.** (A-C) Immunofluorescence staining of muscle sections from WT and *DMD* KO rabbits with mouse monoclonal antibodies against dystrophin (A), glycosylated α-dystroglycan (B) and α-sarcoglycan (C). Nuclei were stained by DAPI. Scale bar: 100 µm.

To examine whether the mutations in the rabbits induced by CRISPR/Cas9 disrupt the expression of the *DMD* gene in the KO rabbits, we performed quantitative reverse transcription PCR (RT-PCR) analysis of *DMD* expression in the gastrocnemius muscle of KO and wild-type (WT) rabbits using two sets of primers. As shown in Fig. S3, the expression of *DMD* was significantly reduced in the skeletal muscle of *DMD* KO rabbits compared with WT controls. To further examine the expression of dystrophin protein in the muscle of these rabbits, we performed immunofluorescence staining. Dystrophin was detected at the sarcolemma of skeletal muscle fibers of WT rabbits, and its expression was completely disrupted in the KO rabbits (Fig. 2A). It has been shown previously that dystrophin forms a large dystrophin-glycoprotein complex (DGC) on the muscle membrane, and dystrophin deficiency would compromise the integrity of the entire DGC. This is also true for DMD KO rabbit skeletal muscle in which the glycosylated form of α-dystroglycan (Fig. 2B) and α-sarcoglycan (Fig. 2C) were greatly reduced. Therefore, our data suggest that engineered mutations in exon 51 lead to disruption of dystrophin and its associated complex.

### Muscular dystrophy presentation in *DMD* KO rabbits

**Fig. 3.**
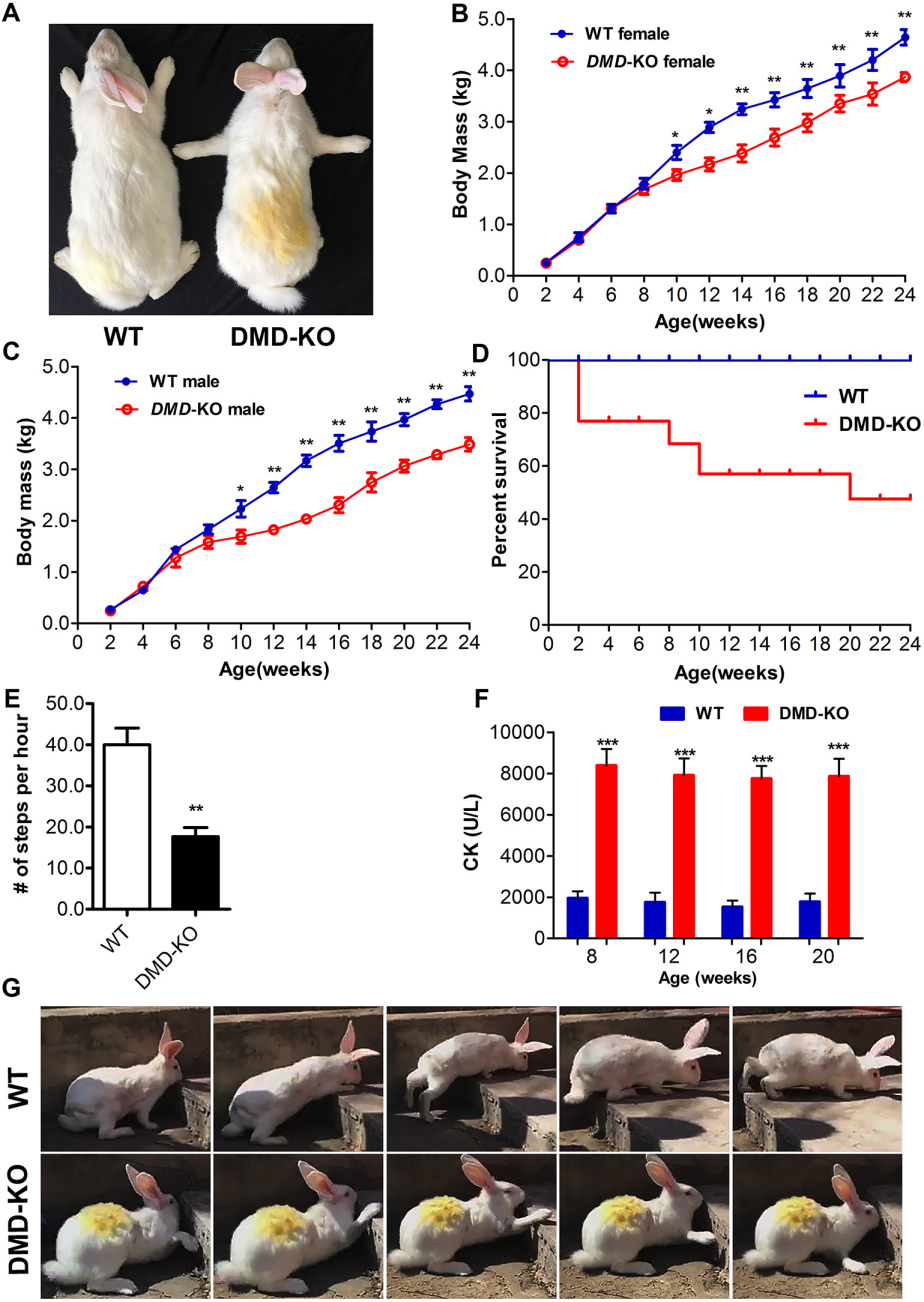
**Impaired physical activity in *DMD* KO rabbits.** (A) Photograph of a 12-week-old *DMD* KO rabbit and its WT littermate showing significant forelimb paralysis in the KO rabbit. (B,C) Body mass comparison of *DMD* KO and WT female (B) and male (C) rabbits from birth to 24 weeks of age (*n*=6). (D) Kaplan–Meier survival curves for the *DMD* KO and WT rabbits (*n*=22). (E) The walking steps during a 1-h recording period for the *DMD* KO and WT rabbits (*n*=6). (F) N-acetyl-L-cysteine measurements of serum CK levels (*n*=6). (G) Photographs of a *DMD* KO rabbit and WT control rabbit performing a stair-climb challenge. *P<0.05; **P<0.01; ***P<0.001.

The *DMD* KO rabbits were smaller in size compared with their WT littermates, and exhibited obvious forelimb paralysis (Fig. 3A). The *DMD* KO rabbits and control littermates were weighed weekly, and the data showed that both the female and male KO rabbits were significantly lighter than their control littermates (Fig. 3B,C). Approximately 20% of the *DMD* KO rabbits died within the first 2 weeks after birth, and close to half (42.6%) died by 20 weeks of age (Fig. 3D).

DMD patients show progressive decline in ambulation, which can be evaluated by a 6-min walk test (6MWT) (McDonald et al., 2010). The 6MWT is also the most accepted primary clinical endpoint in ambulatory DMD trials and has also been used to assess dogs with heart disease (Boddy et al., 2004). To examine the physical activity of the rabbits and to design a simple 6MWT-like assay for preclinical trials using these rabbits, we mounted a human-used activity-monitoring device (Millet Sports Bracelet Wearable Device) on the right hind leg of rabbits and counted the walking steps within 1 h. The KO rabbits showed significantly decreased mobility compared with the healthy ones (Fig. 3E). This 1-h wearable device-assisted walking test (1HWDAWT) provides an extremely simple and inexpensive way to evaluate the physical activity of the freely moving rabbits.

Muscle injury is a hallmark of muscular dystrophy, which releases muscle proteins such as CK into the circulation. We observed that serum CK was significantly elevated in the KO rabbits from 8 to 20 weeks of age compared with their age-matched littermate controls (Fig. 3F). In addition, serum aspartate aminotransferase (AST) and alanine aminotransferase (ALT) were elevated in the DMD KO rabbits (data not shown), which could also be caused by muscle breakdown. When the rabbits were placed in front of a step, WT rabbits easily climbed up the step whereas the KO rabbits failed with obvious attempts (Fig. 3G; Movies 1 and 2).

**Fig. 4.**
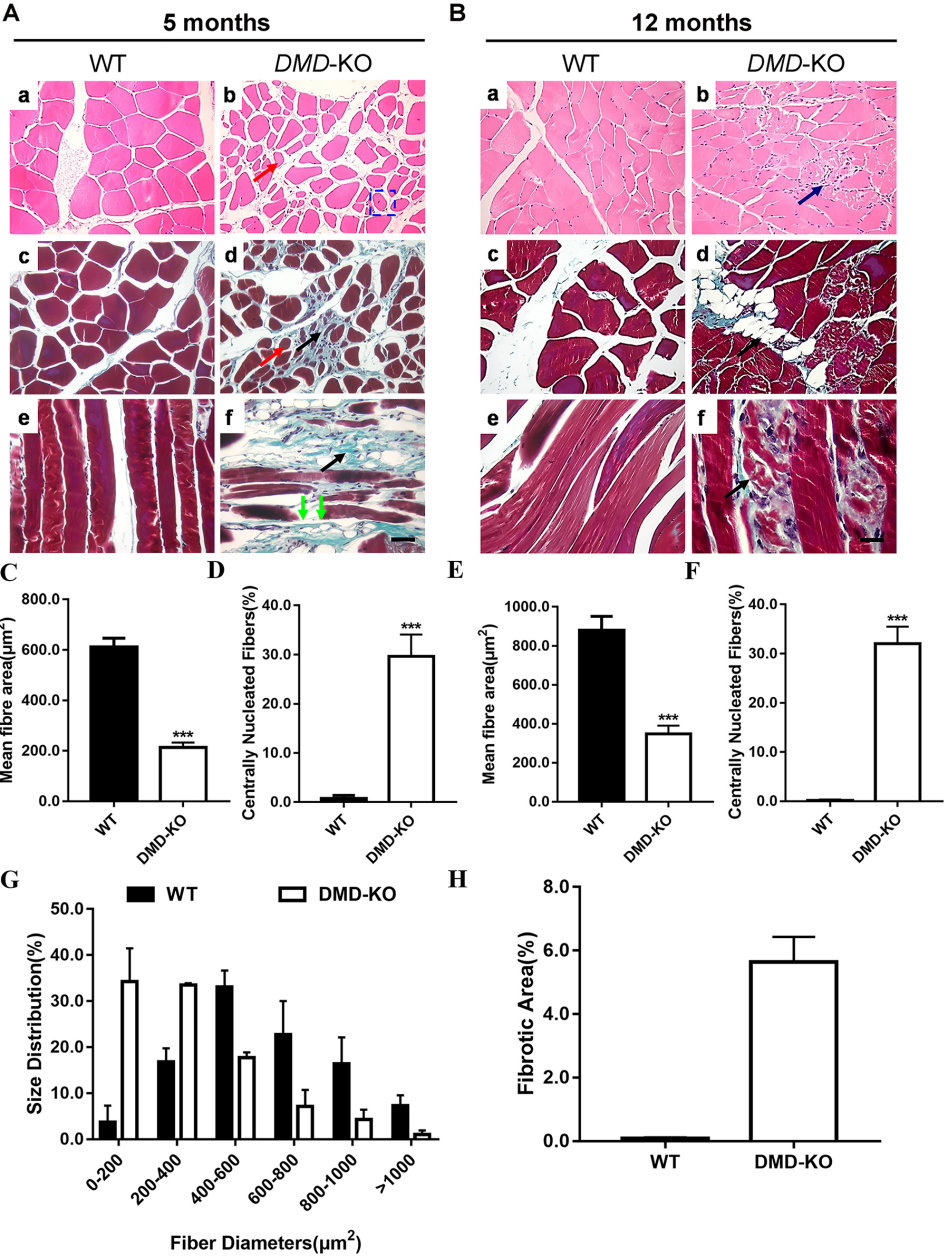
**Muscular dystrophy presentation in *DMD*-KO rabbits.** (A,B) Analysis of H&E- and Masson's trichrome-stained sections of gastrocnemius from 5-month-old (A) and 12-month-old (B) WT and *DMD* KO rabbits. *DMD* KO rabbits displayed myopathy with excessive fiber size variation (red arrows), fiber fracture (green arrows), fibrosis (black arrows) and central nucleated fibers (blue rectangle). (C,E) Quantification of mean gastrocnemius muscle fiber area in WT and *DMD* KO rabbits at 5 (C) and 12 (E) months of age. (D,F) Quantification of centrally nucleated fiber (CNF) percentage in WT and *DMD* KO rabbits at 5 (D) and 12 (F) months of age. (G) Size distribution of WT and *DMD* KO gastrocnemius muscle at 5 months of age. (H) Quantification of relative fibrotic area in WT and *DMD* KO rabbits at 5 months of age. Scale bars: 50 µm. ***P<0.001; *n*=5.

To further examine the histopathology of the *DMD* KO rabbits, we performed Hematoxylin and Eosin (H&E) and Masson's trichrome staining of the gastrocnemius muscle sections from the rabbits at 16 and 20 weeks of age. As shown in Fig. 4, the *DMD* KO rabbits displayed typical muscular dystrophy signs, as evidenced by increased fiber size variation, centrally nucleated fibers, fibrosis and fatty replacement (Fig. 4A,B). The average fiber area was significantly decreased, owing to the cycles of degeneration and regeneration (Fig. 4C,E), and was accompanied by an increased percentage of muscle fibers with central nuclei (Fig. 4D,F). The fiber size distribution (Fig. 4G) showed that there was a significant increase in the smaller fibers, consistent with an increased regeneration in dystrophic muscle. The fibrotic area was significantly increased in the *DMD* KO rabbits compared with WT rabbits (Fig. 4H). These pathological changes in skeletal muscle resemble the typical features of human DMD. Similar pathological alterations were also observed in other muscles, including the tibialis anterior (Fig. S4) and quadriceps (Fig. S5) muscles. The diaphragm muscles, which are severely affected in DMD patients, mice and dogs (Moser, 1984; Valentine et al., 1988), were also found to have extensive muscle degeneration and fibrosis (Fig. S6).

### Cardiomyopathy in the *DMD* KO rabbits

**Fig. 5.**
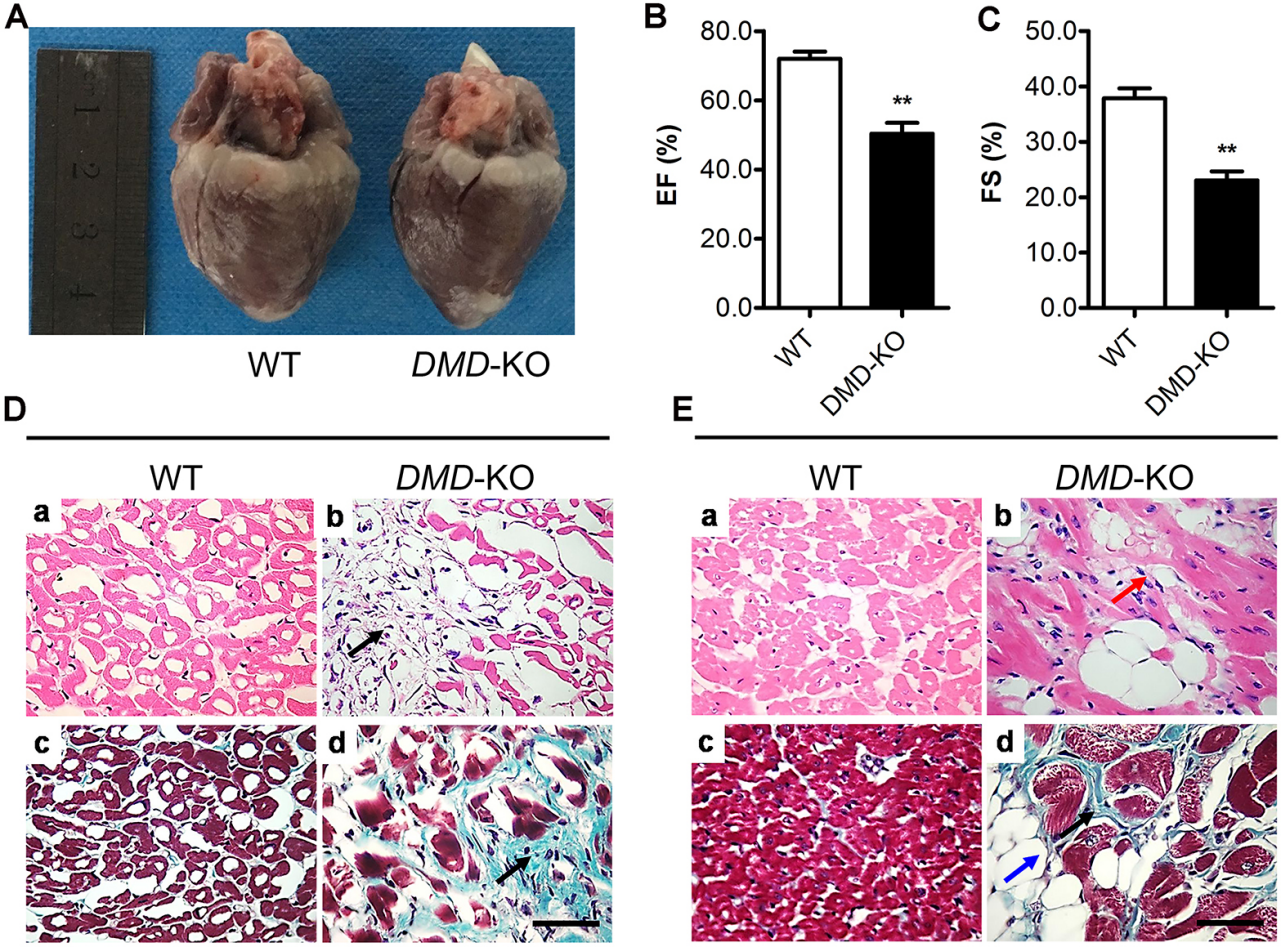
**Cardiomyopathy in the *DMD* KO rabbits.** (A) The hearts from a *DMD* KO rabbit and WT control at 20 weeks of age. (B,C) The left ventricular ejection fraction (EF, B) and fractional shortening (FS, C) were decreased in *DMD* KO rabbits. (D,E) H&E-stained and Masson's trichrome-stained sections of cardiac muscle from WT and *DMD* KO rabbits at 5 (D) and 12 (E) months of age showed significant fibrosis (black arrows), mononuclear inflammatory cell infiltration (red arrow) and adipose tissue (blue arrow). Scale bars: 50 µm. **P<0.01; *n*=5.

Approximately 95% of DMD patients develop cardiomyopathy by 20 years of age, and 20% of these patients die from cardiac complications (Muntoni, 2003; Shirokova and Niggli, 2013). The *Mdx* mice, a widely used animal model for DMD, do not show obvious cardiac pathology until a year later (Pastoret and Sebille, 1995), making it problematic for studying the DMD-associated cardiomyopathy. To examine whether disruption of *DMD* in rabbits causes any pathology in the heart, we performed histological and functional assessments of these rabbits. At 16 weeks of age, the hearts from the *DMD* KO rabbits were grossly similar in size to those from the WT controls (Fig. 5A). We measured cardiac function by echocardiography recording at 4 months of age. The left ventricular ejection fraction (EF) and fraction shortening (FS) of the *DMD* KO rabbits were significantly decreased compared with those of the control rabbits (Fig. 5B,C). Consistent with the detected cardiac dysfunction, the *DMD* KO rabbits also exhibited significant loss of cardiomyocytes and increased interstitial fibrosis, as shown by H&E and Masson's trichrome staining (Fig. 5D,E). These results suggest that the *DMD* KO rabbits developed cardiomyopathy.

## DISCUSSION

In this study, we generated a novel rabbit model for DMD via the zygote injection of Cas9 mRNA and a pair of sgRNAs targeting exon 51 of the *DMD* gene, and demonstrated that the DMD KO rabbits exhibited almost all hallmarks of the disease observed in DMD patients, including muscular dystrophy, cardiomyopathy and high incidence of premature death. To the best of our knowledge, this is the first report of a DMD rabbit model that develops both muscular dystrophy and cardiomyopathy.

Approximately 74% of the live rabbit pups carried mutations at the target sites of the *DMD* gene and ∼85% of these targeted animals carried biallelic mutations. Many of these mutations are small deletions or insertions, disrupting the reading frame of the *DMD* gene. Sanger sequencing and T7E1 assay did not detect significant off-target activities at the top predicted off-target sites. These results are consistent with previous reports that cytoplasmic injection of sgRNA-directed CRISPR/Cas9 mRNA can be used as an efficient approach to generate targeted KO animals, including rabbits, with high fidelity (Honda et al., 2015; Yuan et al., 2016).

Previous studies have shown the *DMD* mutation hotspots are located in the regions of exons 3-7 and exons 45-55 (Badalian and Malygina, 1995; Koenig et al., 1989). We thus chose exon 51 as the target site. Small deletions/insertions (with the number of nucleotides deleted or inserted indivisible by three) in this exon would cause frameshift and thus disrupt the dystrophin expression. Indeed, we observed that the *DMD* transcript was greatly reduced in the skeletal muscle of *DMD* KO rabbits.

Using serum CK as an indicator of muscle injury, we found that it was significantly elevated in the *DMD* KO rabbits as early as 8 weeks of age. In comparison with WT littermates, *DMD* KO rabbits exhibited biochemical and pathological phenotypes characteristic of human *DMD*, including elevated serum CK levels, muscle degeneration and regeneration, interstitial fibrosis, fatty replacement and mononuclear inflammatory cell infiltration. Various skeletal muscle groups, including gastrocnemius, tibialis anterior, quadriceps and diaphragm, are affected in *DMD* KO rabbits. These observations are similar to those in other animal models of DMD, including mice (Sicinski et al., 1989; Spurney et al., 2009; Tanabe et al., 1986), pigs (Klymiuk et al., 2013; Yu et al., 2016) and dogs (Baltzer et al., 2007; Cooper et al., 1988; Jones et al., 2004; Sharp et al., 1992; Smith et al., 2011). We could not measure the contractility of skeletal muscle from the *DMD* KO rabbits owing to the unavailability of the force measurement setup. It was also not feasible to perform grip strength measurement on these animals owing to their large body size. However, functional defects of skeletal muscle in the *DMD* KO rabbits were obviously detected by the reduced physical activity and impaired ability to climb up the step, very similar to DMD pigs (Klymiuk et al., 2013) and boys with DMD in their early life (Zhu et al., 2013) (Table 3).

**Table 3.**
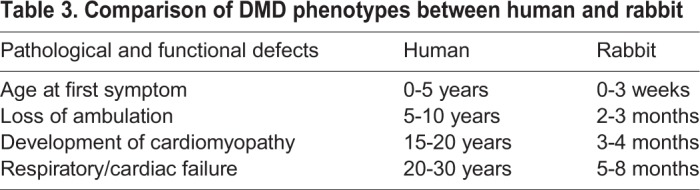
**Comparison of DMD phenotypes between human and rabbit**

Different from *Mdx* mice, the *DMD* KO rabbits showed clear cardiomyopathy at 4 months of age, as evidenced by reduced left ventricular ejection fraction (EF) and fractional shortening (FS) (Fig. 5B,C). Therefore, the *DMD* KO rabbit can be used in preclinical studies to evaluate the therapeutic effects of the tested medicine or treatment on muscular dystrophy and cardiomyopathy. The relatively lower maintenance cost and shorter gestational duration compared with dog and pig models make the *DMD* KO rabbit a particularly attractive model for preclinical studies. We showed that human-used activity-monitoring devices, such as the Millet Sports Bracelet Wearable Device used in this study, can be easily mounted on freely moving rabbits to monitor their physical activities. Moreover, echocardiography recording can be performed to assess their cardiac function.

To the best of our knowledge, this is the first report of a *DMD* rabbit model with close resemblance to its human counterpart. This new model could facilitate basic research to understand the pathogenesis of DMD, and translational studies to develop novel therapeutic strategies for this devastating disease.

## MATERIALS AND METHODS

### Animals and ethics statement

The New Zealand rabbits used in this study were maintained at the Laboratory Animal Center of Jilin University. All experiments involving rabbits in this study were performed in accordance with the guide of the Animal Care and Use Committee of Jilin University.

### CRISPR/Cas9 sgRNA preparation, embryo microinjection and embryo transfer

The CRISPR/Cas9 sgRNA was designed and assembled as previously described (El Refaey et al., 2017; Xu et al., 2015b; Xu et al., 2016). The annealed sgRNA oligonucleotides were cloned into the *Bbs*I sites of pUC57-T7-sgRNA cloning vector (Addgene ID 51306) as described (Shen et al., 2013). The vector of pUC57-T7-sgRNA was PCR amplified using T7 primers (Forward, 5′-GAAATTAATACGACTCACTATA-3′; Reverse, 5′-AAAAAAAGCACCGACTCGGTGCCAC-3′), and the PCR products were transcribed *in vitro* with a MAXIscript T7 Kit (Ambion; Applied Biosystems, CA, USA) and purified using a miRNeasy Mini Kit (Qiagen, Hilden, Germany) according to the manufacturer’s instructions.

The 3xFLAG-NLS-SpCas9-NLS vector (Addgene ID 48137) (containing Cas9 from *Streptococcus pyogenes*), was linearized with *Not*I and transcribed *in vitro* using a mMessage mMachine SP6 Kit (Ambion) and RNeasy Mini Kit (Qiagen) according to the manufacturer's instructions.

The microinjection procedure and embryo transfer were performed essentially the same as previously described (Sui et al., 2016). Briefly, female New Zealand White rabbits at the age of 6-8 months were superovulated with 50 IU follicle stimulating hormone (FSH) at intervals of 12 h six times, mated with male rabbits, and injected with 100 IU human chorionic gonadotropin (HCG). The female rabbits were then euthanized and the oviducts were flushed with 5 ml Dulbecco's phosphate-buffered saline (DPBS)-bovine serum albumin (BSA) for zygote collection. Rabbit embryos were collected at the pronuclear stage. A mixture of *in vitro*-transcribed Cas9 mRNA (200 ng/µl) and sgRNA (50 ng/µl) was microinjected into the cytoplasm of zygotes. The injected embryos were transferred into Earle's balanced salt solution (EBSS) medium and cultured at 38.5°C in 5% CO_2_ for 20-30 min. Approximately 30-50 injected zygotes were then transferred into the oviducts of recipient rabbits.

### Mutation detection in pups by PCR and sequencing

The genomic DNA from *DMD* KO and WT rabbits was extracted from a small piece of ear tissue using a TIANamp Genomic DNA Kit (Tiangen, Beijing, China) according to the manufacturer's instructions. The sgRNA target sites were amplified by PCR using primers (Forward, 5′-TAGTTTGGCTCAGATTGTAG-3′; Reverse, 5′-AGAATAGACAAAGCAGTGTG-3′). The PCR products were gel purified and cloned into pGM-T vector (Tiangen). A minimum of 14 positive clones were sequenced and analyzed using DNAman.

### T7E1 cleavage assay

The T7E1 cleavage assay was performed as described previously (Xu et al., 2015b, 2013). Briefly, the PCR products as described above were purified, denatured and then re-annealed in NEBuffer 2 (NEB) using a thermocycler. Hybridized PCR products were digested with T7 endonuclease 1 (M0302L, NEB) for 30 min at 37°C and subjected to 2% agarose gel electrophoresis.

### Off-target analysis

The top off-target sites were predicted using the online CRISPR Design tool developed by the Zhang group at Massachusett's Institute of Technology (http://crispr.mit.edu/). The PCR products for these potential off-target sites using the primers listed in Table S1 were subjected to T7E1 assay and Sanger sequencing.

### RNA extraction, RT-PCR and quantitative RT-PCR

Total RNA was isolated from the gastrocnemius muscle of WT and *DMD* KO rabbits using TRNzol-A+ reagent (Tiangen), and treated with DNase I (Fermentas Inc., MD, USA). The first-strand cDNA was synthesized using the cDNA first strand synthesis kit (Tiangen). The cDNA was used for regular RT-PCR and quantitative RT-PCR (qRT-PCR) analyses to examine the expression of DMD. The primers were used for regular RT-PCR (Forward, 5′-GTCAACTATCTACTGCAAGAGC-3′; Reverse, 5′-CTGTACTTCATCCCACTGATTC-3′) and qRT-PCR (Forward, 5′-CCGAACTAGAAATGCCATCTT-3′; Reverse, 5′-CACAATCACTTGCTGCGATTT-3′). qRT-PCR was performed using a BioEasy SYBR Green I Real Time PCR Kit (Bioer Technology, Hangzhou, China), and the 2^−ΔΔCT^ formula was used to analyze gene expression, *Gapdh* was used as a reference gene. All experiments were repeated three times for each gene. Data were expressed as the mean±s.e.m.

### Body weight and survival curve

The body weight of age- and sex-matched WT and *DMD* KO rabbits were measured biweekly. All data were expressed as the mean±s.e.m., and a minimum of three individual animals of each genotype were used in all experiments.

### Serum biochemistry analysis

The blood samples were collected into heparinized tubes from the ear vein, and sera were prepared by precipitation and centrifugation. Serum CK, ALT and AST levels were measured using a CK test kit (N-acetyl-L-cysteine method), ALT test kit (continuous monitoring method) and AST test kit (continuous monitoring method), respectively (Ningbo Ruiyuan Biotechnology Co., China).

### Activity measurement

A Millet Sports Bracelet Wearable Device was used to record movement steps within a 1-h period for *DMD* KO and WT rabbits. The rabbits wearing the device on their right hind leg were placed in an ∼30 m^2^ room to allow free movement.

### Echocardiography

Echocardiography recording was performed as described previously (Han et al., 2007; Xu et al., 2015a). Briefly, two-dimensional and M-mode transthoracic echocardiography were performed as previously described on WT and *DMD* KO rabbits (*n*≥3 per group) by an SIUI all digital color doppler ultrasound diagnostic system (Apogee 300, ShanTou, China). Rabbits were studied in right lateral recumbency from parasternal long and short axis views. The rabbits were held in the right position by restraining their limbs with people. A linear array probe and center frequency of 10.0 MHz were used. Cardiac dimensions [the interventricular septal thickness at end-diastole (IVSd), left ventricular end diastolic diameter (LVDd) and left ventricular systolic diameter (LVDs)] were determined and the percentage of FS and left ventricular EF calculated.

### Histology analysis

Various tissues, including gastrocnemius, tibialis anterior, quadriceps, diaphragm and heart, were collected from *DMD* KO and WT rabbits (euthanized at 5 and 12 months of age). The tissues were fixed in 4% paraformaldehyde at 4°C, dehydrated in increasing concentrations of ethanol (70% for 6 h, 80% for 1 h, 96% for 1 h and 100% for 3 h), cleared in xylene and embedded in paraffin for histological examination. The 5-μm sections were cut for H&E (Han et al., 2007; Xu et al., 2015a) and Masson's trichrome staining as previously described. The stained sections were imaged with a Nikon TS100 microscope.

### Immunofluorescence staining

Paraffin-embedded skeletal muscle tissues were deparaffinized and antigen retrieval was performed using a pressure cooker. After blocking with 10% goat serum in PBS for 1 h at room temperature, the tissue sections were incubated with primary antibodies diluted in PBS with 1% BSA at 4°C overnight. Primary antibodies against dystrophin (MANDYS1 clone 3B7, 1:20, Developmental Studies Hybridoma Bank), α-dystroglycan (sc-53987, 1:50, Santa Cruz Biotechnology), α-sarcoglycan (NCL-L-a-SARC, 1:100, Leica Biosystems) and caveolin 3 (610420, 1:500, BD Biosciences) were used. The slides were then extensively washed with PBS and incubated with secondary antibodies (Alexa Fluor 594-conjugated goat anti-mouse IgG, 1:500, Invitrogen) for 1 h at room temperature. Finally, the glass slides were mounted using VECTASHIELD Mounting Medium with 4′,6-diamidino-2-phenylindole (DAPI) (Vector Laboratories). Then the slides were imaged with a Nikon Ti-E inverted fluorescence microscope equipped with an Andor Zyla sCMOS camera and a Nikon Super Fluor 20×/0.75 NA objective lens. Images were recorded using the NISElements Advanced Research software package (Nikon) and processed using Photoshop CS5 (Adobe).

### Morphometric analysis of myofibers

The H&E-stained cross-sections of gastrocnemius, tibial anterior and quadriceps muscles from the *DMD* KO and WT rabbits at 5 months of age were analyzed for fiber size, central nucleation and fibrosis. A minimum of three different regions were counted per section. The fiber size, percentage of central nucleated fibers and fibrotic area were calculated using ImageProPlus 6.0 software (Media Cybernetics, Silver Spring, MD, USA).

### Statistics

Data are expressed as mean±s.e.m. Statistical differences were determined by unpaired Student's *t*-test for two group comparisons, and one-way ANOVA with Bonferroni's post-tests for multiple group comparisons, using Prism 7.0 (GraphPad). *P*≤0.05 was considered significant.

## Supplementary Material

Supplementary information

First Person interview
