# Supplementary material for: A novel rabbit model of Duchenne muscular dystrophy generated by CRISPR/Cas9
Source: Dis Model Mech. 2018 Jun 4;11(6):dmm032201. doi: 10.1242/dmm.032201 (PMC6031364; doi:10.1242/dmm.032201)
Supplement: Supplementary information [file dmm-11-032201-s1.pdf]

The Company of Biologists

FIRST PERSON

First person – Tingting Sui

First Person is a series of interviews with the first authors of a selection of papers published in Disease Models & Mechanisms, helping early-career researchers promote themselves alongside their papers. Tingting Sui is first author on 'A novel rabbit model of Duchenne muscular dystrophy generated by CRISPR/Cas9', published in DMM. Tingting is a PhD student in the labs of Liangxue Lai and Zhanjun Li at Jilin University, China, focusing on the development of rabbit models for neuromuscular degenerative diseases.

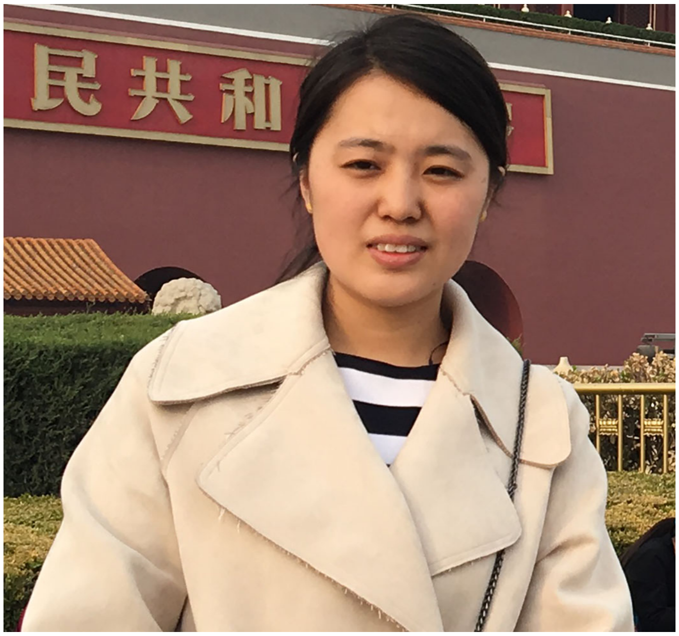

Tingting Sui

How would you explain the main findings of your paper to non-scientific family and friends?

Duchenne muscular dystrophy (DMD) is a lethal muscle disease, caused by genetic defects in the gene encoding the protein dystrophin on the X chromosome. Previous research into the causes and physiology of the disease, as well as preclinical studies of potential treatments, has primarily been conducted in mice. However, the translational potential of the findings in mice to humans has been unsatisfactory, partly due to the differences in physiology between these two species. Although large animal models of DMD including dog and pig are available, their use is expensive and limited to only a few facilities around the world. Therefore, we engineered a rabbit model of DMD using embryonic genome editing. Our data shows that the DMD rabbits exhibit many of the typical signs of muscular dystrophy seen in DMD patients, including severely impaired physical activity, elevated serum creatine kinase levels and progressive muscle loss.

What are the potential implications of these results for your field of research?

We believe that our rabbit model of DMD has great potential to facilitate the basic research that furthers our understanding of the pathogenesis of DMD, and translational studies to develop novel therapeutic strategies for this devastating disease.

What are the main advantages and drawbacks of the model system you have used as it relates to the disease you are investigating?

The rabbit model of DMD has several advantages. The DMD rabbits recapitulate human DMD better than mouse models, in particular regarding lifespan and the development of cardiomyopathy. Both the model rabbits and patients with DMD develop dilated cardiomyopathy in their early life (roughly at puberty) while the commonly used mdx mice show very mild cardiac defects after a year. The DMD model rabbits also exhibit the shortened lifespan of individuals with DMD, while mdx mice have almost a normal lifespan. Additionally, the maintenance cost for rabbits is significantly lower and gestational duration is also very favorable for research laboratories, in comparison to large animal models of DMD. All of these features make the rabbit model a good choice to study human DMD.

Tingting Sui's contact details: Jilin Provincial Key Laboratory of Animal Embryo Engineering, Jilin University, Changchun 130062, China.

E-mail: suitingting2012@163.com

What has surprised you the most while conducting your research?

It is quite amazing that genetic defects in the gene encoding dystrophin can have such a devastating impact on muscle and heart function in both rabbits and humans. Therefore, I am eager to learn more about this genetic disease and hopefully some day we can develop an effective therapy that can eradicate the disease completely.

> **"The most significant challenge is to precisely correct the mutant genetic code in the whole body."**

Describe what you think is the most significant challenge impacting your research at this time and how will this be addressed over the next 10 years?

Recently, several groups including our collaborator Dr Renzhi Han have demonstrated the great promise of genome editing therapy for DMD. However, the current approach for genome editing therapy of DMD is to slice out the mutant exons or disrupt the splicing site so that the mutant exons can be skipped. Although promising, such a therapy still does not produce wild-type dystrophin protein. The most significant challenge is to precisely correct the mutant genetic code in the whole body. CRISPR/Cas9 technology has evolved quickly and so-called 'base editors' have been engineered, although

Disease Models & Mechanisms

further developments are needed to make more precise and smaller versions so that they can be used *in vivo*.

What changes do you think could improve the professional lives of early-career scientists?
I think it is very important for early-career scientists to get exposure to cutting-edge research. Attending scientific conferences, holding seminars for invited speakers, and constructing an effective communication network of leading scientists are all good ways to foster the growth of early-career scientists.

What's next for you?
My current goal is to develop rabbit models of neuromuscular degenerative diseases, which would be used as a valuable resource for understanding the pathophysiological mechanisms of and developing novel therapies for these diseases.

References
Sui, T., Lau, Y. S., Liu, D., Liu, T., Xu, L., Gao, Y., Lai, L., Li, Z. and Han, R. (2018). A novel rabbit model of Duchenne muscular dystrophy generated by CRISPR/Cas9. *Dis. Model. Mech.* **11**: dmm032201.
